# Low-Wage Agricultural Migrant Workers in Apulian Ghettos, Italy: General Health Conditions Assessment and HIV Screening

**DOI:** 10.3390/tropicalmed6040184

**Published:** 2021-10-15

**Authors:** Francesco Di Gennaro, Rossana Lattanzio, Carmine Falanga, Silvia Negri, Roberta Papagni, Roberta Novara, Gianfranco Giorgio Panico, Valentina Totaro, Mariacristina Poliseno, Davide Fiore Bavaro, Lucia Raho, Marcella Schiavone, Nicole Laforgia, Alessandro Volpe, Renato Laforgia, Sergio Lo Caputo, Claudia Marotta, Giovanni Putoto, Annalisa Saracino

**Affiliations:** 1 Clinic of Infectious Diseases, University of Bari, University Hospital Policlinico, 70124 Bari, Italy; rossana.lattanzio@gmail.com (R.L.); robertapapagni0@gmail.com (R.P.); roberta.novara@gmail.com (R.N.); panico.gianfranco@gmail.com (G.G.P.); valenduzza@hotmail.it (V.T.); davidebavaro@gmail.com (D.F.B.); alessandro_volpe@hotmail.it (A.V.); annalisa.saracino@uniba.it (A.S.); 2 Operational Research Unit, Doctors with Africa CUAMM, 35121 Padua, Italy; luxraho@gmail.com (L.R.); marcella.schiavone@gmail.com (M.S.); n.laforgia@gmail.com (N.L.) renatolaforgia@gmail.com (R.L.); marotta.claudia@gmail.com (C.M.); g.putoto@cuamm.org (G.P.); 3 ANLAIDS Sezione Lombarda, 20124 Milano, Italy; falanga@anlaidslombardia.it (C.F.); psico.silvianegri@gmail.com (S.N.); 4 Infectious Diseases Unit, Department of Clinical and Experimental Medicine, A.O.U. “Policlinico Riuniti”, 710121 Foggia, Italy; polisenomc@gmail.com (M.P.); sergiolocaputo@gmail.com (S.L.C.); 5 Italian Society for Infectious and Tropical Diseases—(SIMIT), Appulo Lucana Section, 70124 Bari, Italy

**Keywords:** migrant, health status, HIV, Puglia

## Abstract

Background: Approximately 500,000 migrants work in the agricultural sector in Italy. Many of them live in shantytowns, wrongly called “ghettos”, far away from city centers, with no water, proper hygienic conditions or health services. The aim of this study is to assess general health conditions and HIV prevalence by giving hygienic and sanitary sustenance. Methods: Between June 2019 and February 2020, we performed a screening campaign for HIV–diabetes–hypertension, involving migrants living in three Apulian establishments: ghetto Pista, “Sankara House” and “Arena House”. Results: Overall, 321 migrants were enrolled in the study. In the medical screening, one HIV test resulted positive. Hypertension was found in 12% of the migrants visited, diabetes in 2% and TB symptoms in 17%. Among others symptoms explored, muscle and joint pain/fatigue resulted in being the most frequent, and was reported by 34% of the migrants, followed by cough (10%). Significant predictors of muscle and joint pain/fatigue were: low BMI values (OR = 1.32; 95% CI 1.19–1.99), the absence of education (OR = 1.85; 95% CI 1.02–2.95), being employed with a regular contract (OR = 2.64; 95% CI 2.39–2.83) and living in the ghettos since >12 months (OR = 1.74; 95% CI 1.24–2.21). Conclusions: Our experience suggests that, in this population, the health condition is mainly linked to the specific working activities in the agricultural fields, as well as to the hygienic and living conditions, and that all of this is due to the lack of social protection in their life and job.

## 1. Introduction

Although there is a lack of official and accurate data, it is estimated that about 500,000 migrant workers are involved in the agricultural sector in Italy; this represents half of all workers employed in agriculture in Italy [1,2].

They are a class of exploited workers and are often immigrants from the poorest countries. Most of the workers indeed come from sub-Saharan Africa, but also from Asia and Eastern Europe. Their living conditions are miserable [2,3]. Agricultural workers are paid on a “piecework” basis, so based on the amount of harvest rather than on the time spent working, like other jobs. Thus, often they are paid more or less 12 euros for eight hours of work under the supervision of corporals [3,4].

These workers work in extremely vulnerable conditions and are further at risk when they are employed illegally by mafia-like organizations. All of this characterizes a reality made by slavery, violence, illegal recruitment, hard work and the total absence of workers’ rights. This phenomenon is called “caporalato”, which is a form of illegal recruitment and exploitation of labor through an intermediary, precisely called “caporale”. It is widespread throughout Italy and it is particularly frequent in the agricultural and farming sectors [3,4]. There are “ringleaders”, the so-called “Caporali”, who are modern-day slave masters. Moreover, workers are often victims of physical assault and sexual violence and the withholding of wages and documents, and all of these are associated with threats to their families if they refuse to work under conditions imposed by the corporal. The corporal has contact with the landowner and is responsible for the transport. The workers are transported from the ghetto to the field through a minibus tested for 10 people, but they are also transported in 30. They arrive at the camp and have to recover a chest that, when empty, already weighs 70 kg. They fill it with tomatoes or oranges or other fruit depending on the season, for a total weight of 400 kg. In order to fill it up, it takes around an hour and a half. One single filled chest pays EUR 1.50 to the migrants. An average, young and healthy migrant, in one day, manages to fill eight boxes in 10 h for a total of EUR 12. Out of these EUR 12, EUR 5 must be subtracted for transport to be delivered to the corporal. A sandwich plus a small bottle of water costs EUR 3.50, and gloves cost EUR 1.50. At the end of the day, EUR 2.00 remain. Out of which, sometimes, 50 cents are subtracted in order to recharge the phone, and EUR 1.00 is substracted for a hot shower. This is money that is always requested and collected by the caporale [2,3,4,5].

Many of them live in ghettos and shanty towns, isolated from urban centers, where houses are made of tents, shacks, sheets and boards, and where windows are made of car wheel rims or seat covers. Those who are luckier live in houses that have been abandoned for a long time, with no water and no heating, which is why they often light fires, which then leads to large and sometimes destructive fires. Hygienic conditions or health services are totally absent [2].

Refs. [3,4,5] It is estimated that there are 50–70 ghettos in Italy that host approximately 100,000 underpaid migrant workers [4,5,6]. This is only an estimate; there is no official census. In spite of the universal health care system and laws protecting migrants’ health, they have limited or no access to essential medical care. Regional governments, NGOs (non-governmental organization), associations and Caritas (social organizations of the Catholic Church) are trying to limit the damage of this huge and currently uncontrolled phenomenon.

Since 2015, Doctors with Africa CUAMM aims to improve the health conditions of agricultural workers living in three ghettos in Puglia by offering free-of-charge primary health care service in several settlements through a mobile clinic and a multidisciplinary team. In order to improve the sanitary assistance, this study aims to assess general health conditions and HIV prevalence.

## 2. Materials and Methods

### 2.1. Study Setting, Design and Population

**Figure 1:**
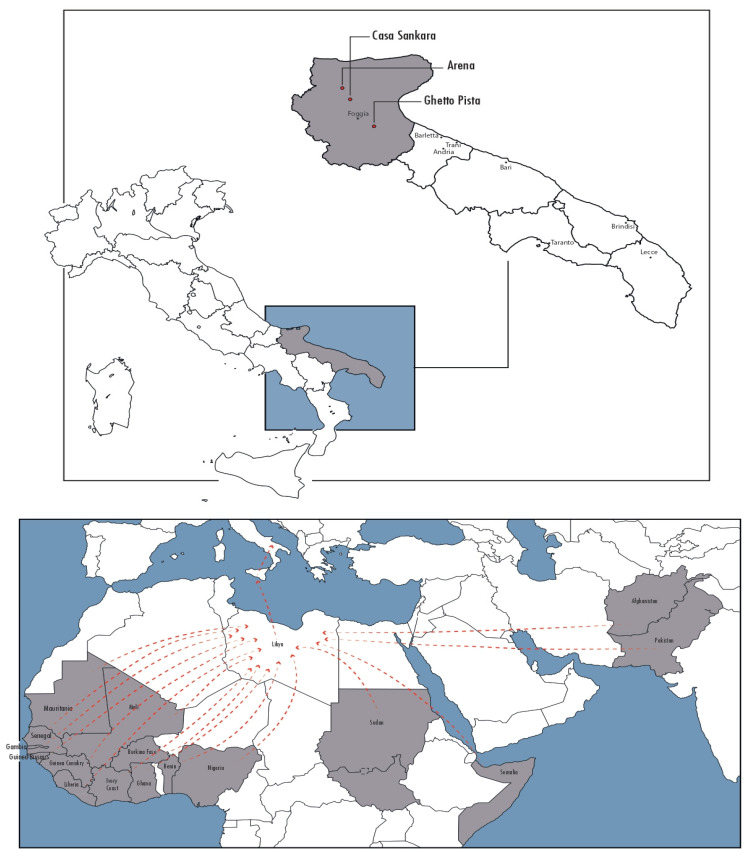
Localization of ghettos in Apulia region and countries of origins of migrants.

Since 2015, Doctors with Africa CUAMM is committed to improving the health conditions of agricultural workers by offering free-of-charge primary health care service in several settlements through a mobile clinic and a multidisciplinary team with at least one specialized doctor, a dentist, two nurses, a cultural mediator, several volunteers and a logistician. As part of the mobile clinic services, between June 2019 and February 2020, Doctors with Africa CUAMM, in partnership with University of Bari-Infectious Diseases Clinic Bari, Anlaids and the Apulian section of the Italian Society for Infectious and Tropical diseases—(SIMIT), performed a screening campaign for HIV–diabetes–hypertension, involving migrants living in three Apulian establishments (Figure 1):

1. Ghetto Pista in Borgo Mezzanone, province of Manfredonia, an informal spontaneous settlement with 1500 estimated inhabitants;
2. “Casa Sankara” and “Arena”, both based in San Severo, province of Foggia, and arranged by the Apulia region for the agricultural workers population.

The eligible population included all of the people who were present in these establishments during the period of study. No exclusion criteria were used for this study.

#### 2.1.1. Questionnaires

A standardized questionnaire was administered through a face-to-face interview conducted by trained nurses and doctors. It is made of questions divided into four sections: (I) socio-demographic information (age, education, occupation, marital status, typology of work contract, document to stay in Italy, information on travel from their country to Italy), (II) sexual habits (possible pregnancy, concurrent sex partners, condom use, smoke habit and alcohol abuse, etc.), (III) medical history (including TB symptoms and other communicable—non communicable diseases) and (IV) information on HIV (previous test, HIV stigma). Before starting the interview, informed consent was obtained and the study aims were explained, as well as the methods used, to ensure the confidentiality of the data. At the end of the interview, participants received health advice if requested. The collected data were entered in a dedicated database and a quality control check of the data entry was performed before data analysis.

#### 2.1.2. Medical Examinations

Medical examinations were carried out and medical treatment was prescribed and issued when needed, and an HIV test was also performed. A basic physical examination (vital signs, weight, height, waist circumference, blood pressure, oxygen saturation and general appearance) was performed. The body mass index (BMI) was calculated and blood pressure (BP) was measured (high pressure defined as BP > 140/90 mmHg). For the diabetes screening, two consecutive fasting blood glucose tests were performed to each participant. According to the WHO guidelines, patients were considered as non-diabetic if both measurements were ≤110 mg/dL and as diabetic if both measurements were above 126 mg/dL. If at least one value was between 110 and 126 mg/dL, the oral glucose tolerance test (OGTT) was performed: patients were considered diabetic when plasma glucose at 2 h was ≥200 mg/dL.

After informed consent, a 3rd generation capillary HIV blood rapid test was used (Alere Determine, Abbott) for HIV testing.

### 2.2. Statistical Analysis

Descriptive analysis was performed to define the distribution of the characteristics of the sample, and a χ^2^ test (with Fisher’s correction if fewer than five cases were present in a cell) was applied for categorical variables. A logistic regression model was implemented as follows. Muscle and joint pain/fatigue was considered as dependent variables and each one of the available factors at the baseline evaluation as independent variables (univariate analysis). In the multivariate analysis, factors with a *p*-value < 0.10, as assessed by univariate analysis, were included. Multicollinearity among covariates was assessed through the variance inflation factor, taking a value of 2 as cut-off to exclude a covariate. However, no variables were excluded according to this pre-specified criterion. Odds ratios (O.R.s) as adjusted odds ratios (adj-O.R.s) with 95% confidence intervals (CIs) were used to measure the strength of the association between factors at the baseline (exposure) and presence of muscle and joint pain/fatigue (outcome). All statistical tests were two-tailed and statistical significance was assumed for a *p*-value < 0.05. Statistical analyses were performed with GraphPad Prism version 8.0 (GraphPad Software, Inc., San Diego, CA, USA).

## 3. Results

From June 2019 and February 2020, 321 migrants (n 298, 92% male, mean age 29 years IQR 18–56) were enrolled in the study. Fifty percent (n 162) were living in the ghetto Pista, whereas the other half was based in the structures arranged by the Apulian region (n 134, 42% in “Casa Sankara” and 25, 8% in “Arena”). Forty-one per cent (n 131) of the all sample was made by migrants from Senegal, followed by 24% (n 78) from Gambia (Figure 1). Most of the migrants interviewed (n 305, 95%) reported a stopover in Libya during their trip toward Italy. Thirty-nine per cent of the sample (n 125) declared to be married, while 59% (n 191) declared to have children. When exploring their current occupation, 83% (n. 264) reported to be an agricultural worker, 3% (n. 9) to be a sex worker and 8% (n. 28) to be unemployed. Forty-three per cent (n. 139) declared to have a regular employment contract. Overall, the mean time of their stay in Italy was of 55 months. Eleven per cent of the migrants enrolled in the study declared to have a family doctor. Other socio-demographic information collected are reported in Table 1. Health perceptions and behaviors reported during the interview are reported in Table 2. Overall, at the medical screening, one HIV test resulted positive. Hypertension was found in 12% (n. 40) of the migrants visited, tachycardia in 4% (n. 13), diabetes in 2% (n. 6), hypoxemia in 4% (n. 14), TB symptoms in 17% (n. 53) and genital secretions/ulcerations in 2% (n. 5) (Table 3). Among others symptoms explored, muscle and joint pain/fatigue resulted in being the most frequent, being reported by 34% (n. 110) of the migrants, followed by cough (n. 31, 10%) and headache (n. 26, 8%). Significant predictors of muscle and joint pain/fatigue were: low BMI values (OR = 1.32; 95% CI 1.19–1.99), absence of education (OR = 1.85; 95% CI 1.02–2.95), being employed with a regular contract (OR = 2.64; 95% CI 2.39–2.83) and living in the ghettos for >12 months (OR= 1.74; 95% CI 1.24–2.21) as showed in Table 4.

## 4. Discussion

Our study presents the results of a health screening performed in agricultural migrant workers living in one ghetto and two establishments arranged by the region, in Apulia. In our sample, a low prevalence of HIV, diabetes and hypertension was found, whereas the signs and symptoms of their heavy working conditions and low quality of life clearly emerged. In fact, even though the majority of the migrants interviewed came to Europe from African and Asian countries with the hope of improving their economic status and quality of life, living in ghettos and working in the agricultural sector without any social protection could not live up to their expectations.

Almost all migrants enrolled were men and young (median age of 29 years), and many of them declared that they had been in the ghetto for at least one year and a half in a total period of four years in Italy. Only half of the sample claimed to have legitimate documents to reside in Italy, and less than half reported to have a regular job contract. Three to up to four migrants interviewed reported that they had never been tested for HIV and, notably, the vast majority did not use condoms during sexual intercourse, despite only one migrant testing positive. In terms of non-communicable diseases, 12.5% had hypertension, whereas just 2% had diabetes. These numbers are lower compared to data collected on population of migrants from North Africa in Italy [7]. While the most interesting finding is about sanitary situation related to work, in fact, three quarters of the sample reported signs and symptoms associated with outdoor labor (muscular pains, fatigue, headache, asthenia, etc.).

Interestingly, analyzing our data, a low BMI, no education, no regular contract and a long stay in the ghettos resulted in being risk factors for muscle and joint pain/fatigue. These underline how living in the ghettos with a poor quality of life and no job rights recognized through a regular employment contract could be strongly related to health problems and, thus, how social determinants of health are always important in a global health approach [8,9,10].

Few data are available on the health status of migrant workers in Europe, but previous studies in Nepal, Tanzania and Ethiopia have documented a low prevalence of HIV in migrant farm workers, low condom use, high risk of risky sex and high prevalence of malnutrition, and reinforce the need for screening for diseases related to a poor quality of work and life, which is strongly correlated with tuberculosis and non-communicable diseases, such as hypertension and diabetes, and overwork illnesses, such as rickets, asthenia, muscle/fatigue pain and mental health illnesses [11,12,13,14].

To the best of our knowledge, this is the first health assessment on migrants employed in the agricultural sector and living in ghettos in Italy. Indeed, limited and fragmented data on their health condition, and especially on their health needs, are accessible, making national and international policy responses to their health needs more challenging.

Preliminary evidence-based recommendations that can be raised from our findings are to actively search for signs and symptoms of TB and other infectious diseases, including sexually transmitted infections or those endemic to the country of origin (such as malaria, intestinal parasitosis, etc.), but also priority to non-communicable diseases, such as diabetes and hypertension, should be given when promoting a health intervention in such a setting [15,16]. It should also be important to focus on the interaction between non-communicable and communicable diseases in worsening clinical presentations and outcomes [17,18,19]. From our experience, an appropriate use of point of care HIV testing in these settings could also be highlighted, since it could help to reduce stigma and increase adherence to screening [20]. The impact of the COVID-19 pandemic on migrants and refugees’ health should also be assessed. In fact, as other epidemics have already shown, e.g., Ebola, during the pandemic, due to the high pressure on hospitals, many voluntary activities and health support on the migrant population were interrupted or suspended with understandable health consequences on this vulnerable population. An inclusive approach to refugee and migrant health that leaves no one behind during the COVID-19 pandemic should guide our public health efforts [21,22].

In addition, an important aspect of our study was to involve young medical residents by making them discover such a complex and marginal reality as the ghettos [23]. We hope that this will have an impact on their motivation in their professional life, keeping an inclusive view on global health issues and on the health of vulnerable populations (women, migrants, refugees, children, etc.) [24,25].

In our study, we recognize several limitations: first of all, the complexity of the setting, with the absence of census data of the population living in the settlements—there was no sample calculation to assess the prevalence of the diseases, so a convenient sample made by migrants who voluntarily agreed to join was considered. Then, it should be considered that even if all migrants with evocative signs and symptoms of TB were referred to proper health services to confirm the diagnosis, there was no feedback on the follow up, and so a real estimate of the TB prevalence was impossible to perform. In addition, a big missing area in the health assessment performed is the mental health area; in fact, due to the absence of a proper health professional, the psychological and psychiatric aspects were not evaluated. However, this study was a pilot experience to build a picture of health general conditions in this population, and, starting from all challenges raised, we will try to build further stronger research projects in order to study in depth all findings collected.

## 5. Conclusions

In conclusion, our experience as Doctors with Africa CUAMM and global health activists suggests that baseline health conditions of migrants’ workers are quite good, since they are all young and in health to perform heavy work. The majority of health problems came as consequences of the specific working activities in the agricultural fields, or due to their hygienic conditions of life and their conditions of work. Therefore, the issue is: how can we eradicate the causes of these avoidable deaths and diseases? How can we act to fight the exploitation that these workers have to face? A response is necessary and the health sector should express its concerns and make a stand, with a coordinated and multidisciplinary action. The legal, employment and health protection of low-wage agricultural workers and their families is urgently needed. Health, migration, economy, sustainable development and justice are all interlinked facets of our world and we feel a duty for the science and health community to care and to give a voice to the voiceless. We consider a priority to place migrant and refugees’ health in a central role of the national, European and global agenda as a pillar of global health. Only in this way can health, justice and development be authentically sustainable. A multidisciplinary reflection on the strategy needed to improve health conditions in such settings is urgent and is no longer able to be postponed.

## Figures and Tables

**Table 1:** Socio-demographic information of the 321 migrants enrolled in the study.

		N (%)
	Total Migrants	321 (100)
	Male	298 (92)
	Mean age (SD)	29 (SD)
Ghettos	Pista	162 (50)
	Sankara house	134 (42)
	Arena house	25 (8)
Nationality	Senegal	131 (41)
	Gambia	78 (24)
	Nigeria	32 (10)
	Guinea Conakry	18 (6)
	Other	62 (19)
	Stopover in Lybia	305 (95)
	Married	125 (39)
	Children	191 (59)
Occupation	Agricultural workers	264 (83)
	Sex workers	9 (3)
	Unemployed	28 (8)
	Others	20 (6)
	Regular employment contract	139 (43)
Education	<8 y	300 (93)
	>8 y	21 (6)
	Regular document to stay in Italy	184 (57)
	Hold a regular document in the past	196 (61)
	Time of stay in Italy (mean, months)	55.3 (4–140)
	Time of stay in the ghettos (mean, months)	17.9 (3–100)
	Length of the travel	12.2 (7–22)
Religion	Christian	51 (16)
	Muslim	270 (84)
Family doctors	Yes	36 (11)
	No	285 (89)

**Table 2:** Health perceptions and behaviors reported by the 321 migrants enrolled in the study.

		N (%)
Knowledge about sexual transmitted diseases	No	85 (26)
	Yes	236 (74)
Sexual orientation	Heterosexual	321 (100)
Previous HIV Test	Done	80 (25)
	Never done	238 (74)
	Unknown	3 (1)
Do you rate your health as good?	No	106 (33)
	Yes	215 (67)
Any known diseases	No	293 (91)
	Yes	28 (9)
Fair about possible stigma after a positive HIV test	No	166 (52)
	Unknown	4 (1)
	Yes	151 (47)
Condom use	No	205 (64)
	Yes	116 (36)
Sex for money	No	173 (54)
	Yes	148 (46)
At-risk sexual intercourse in the last 3 months	No	191 (60)
	Yes	130 (40)
Sex under drugs or alcohol	No	307 (96)
	Yes	14 (4)

**Table 3:** HIV results and medical assessment performed on 321 migrants enrolled in the study.

		N (%)
HIV test results	Positive	1 (0)
Hypertension	Yes	40 (12)
Tachycardia (FC > 100)	Yes	13 (4)
Diabetes	Yes	6 (2)
Hypoxemia (<94% xx)	Yes	14 (4)
TB symptoms	Yes	53 (17)
Genital secretions/ulcerations	Yes	5 (2)
Other symptoms	Muscle and joint pain/fatigue	110 (34)
	Cough	31 (10)
	Headache	26 (8)
	Miscellaneous (teeth, epigastric pain, etc.)	62 (19)

**Table 4:** Factors associated with presence of muscle and joint pain/fatigue in agricultural migrant workers.

Characteristics	Univariate Analysis	Multivariate Analysis
	O.R.	Adj-O.R.
Age (years)	1.02 (0.98–1.04)	-
Female	0.28 (0.16–0.40)	0.58 (0.47–0.78) *
Low BMI (<18)	1.80 (1.42–2.02)	1.42 (1.18–1.72) *
Contract of work	1.51 (1.43–0.70)	2.64 (2.39–2.83) *
>12 months in the ghetto	1.85 (1.35–2.45)	1.74 (1.24–2.21) *
Document to stay in Italy	0.10 (0.04–0.85)	0.39 (0.08–1.28)
Sex under influence of drugs or alcohol	0.34 (0.10–1.10)	0.95 (0.22–1.73)
HIV status	1.80 (1.50–2.00)	-
Tb symptoms	1.14 (1.08–1.78)	1.74 (1.50–2.03)
Diabetes	0.25 (0.10–0.40)	0.71 (0.28–1.23)

*: statistically significant.
